# Analysis of Tilt Effect on Notch Depth Profiling Using Thin-Skin Regime of Driver-Pickup Eddy-Current Sensor

**DOI:** 10.3390/s21165536

**Published:** 2021-08-17

**Authors:** Mingyang Lu, Xiaobai Meng, Ruochen Huang, Anthony Peyton, Wuliang Yin

**Affiliations:** 1 School of Electrical and Electronic Engineering, University of Manchester, Sackville Street Building, Manchester M13 9PL, UK; xiaobai.meng@northampton.ac.uk (X.M.); ruochen.huang@manchester.ac.uk (R.H.); a.peyton@manchester.ac.uk (A.P.); 2 Faculty of Art, Science and Technology, University of Northampton, Northampton NN1 5PH, UK

**Keywords:** eddy current driver–pickup sensor, surface crack, depth measurement, thin-skin regime, non-destructive testing

## Abstract

Electromagnetic eddy current sensors are commonly used to identify and quantify the surface notches of metals. However, the unintentional tilt of eddy current sensors affects results of size profiling, particularly for the depth profiling. In this paper, based on the eddy current thin-skin regime, a revised algorithm has been proposed for the analytical voltage or impedance of a tilted driver–pickup eddy current sensor scanning across a long ideal notch. Considering the resolution of the measurement, the bespoke driver–pickup, also termed as transmitter–receiver (T-R) sensor is designed with a small mean radius of 1 mm. In addition, the T-R sensor is connected to the electromagnetic instrument and controlled by a scanning stage with high spatial travel resolution, with a limit of 0.2 μm and selected as 0.25 mm. Experiments were conducted for imaging of an aluminium sheet with seven machined long notches of different depths using T-R sensor under different tilt angles. By fitting the measured voltage (both real and imaginary part) with proposed analytical algorithms, the depth profiling of notches is less affected by the tilt angle of sensors. From the results, the depth of notches can be retrieved within a deviation of 10% for tilt angles up to 60 degrees.

## 1. Introduction

Based on the electromagnetic induction method, eddy current testing (ECT) has been widely implemented for interrogating conductive structures [1,2,3,4,5,6,7,8,9]. Compared to other non-destructive techniques, ECT has the merits of high adaptability and sensitivity, which apply to property measurements and inspections of structural integrity [10,11,12,13,14,15,16,17,18,19]. For the measurement of defects in conductive structures, ultrasonic testing is reliable for identifying and locating (deep) defect clusters, but is hampered by defect shielding [20]. In contrast, ECT is more efficient in quantifying surface notches, particularly for the depth of defect or rolling contact fatigue (RCF) [21] in rails. 

Various methods have been used to analyse the response of eddy current inspection of notches, including numerical models, such as finite element (FE) and boundary element (BE) models, as well as the eddy current thin-skin analytical method. For the numerical FE model, based on Maxwell equations with boundary conditions, the A-V form of the Galerkin equation has been proposed for field computations, including the field of magnetic vector potential A and electric scalar potential V [22]. By putting mesh information of all discretised subdomains together, the problem becomes finding a solution of equations with a sparse stiffness matrix. Techniques have been proposed for hastening the solving process using FE A-V form Galerkin methods, including polishing the sparse stiffness matrix, re-ordering and incomplete LU decomposing, optimised initial preconditioner, perturbed matrix, and weakly coupled effect [8,19,23,24,25]. In addition, other FE models, including the use of reduced magnetic vector potential [26] and alternating current field measurement (ACFM) [27], have been utilised to calculate the field and sensor response. For the BE model, Theodoulidis, Poulakis, and Dragogias proposed an improved method for accelerating the computation of impedance for eddy current sensor scanning over narrow cracks [28]. For the analytical method, the eddy current thin-skin regime has been proposed to approximate the impedance for eddy current sensor scanning over long notches [29,30]. The thin-skin method is based on the single-coil setup under high working frequencies, which is valid for a crack depth and length of at least three times the eddy current skin depth. 

Sensor tilt is identified as one of the major sources of noise in eddy current surface inspections [29]. In this paper, to address the unintentional tilt effect on depth profiling of notches, a revised thin-skin algorithm for tilted T-R sensor scanning across a notch has been proposed. To increase the measurement resolution, the coil of the sensor is wound with a small mean radius of 1 mm. Experimental measurements have been carried out for the voltage mapping of the T-R sensor scanning over the cracks in an aluminium sheet under different tilt angles. To ensure that the thin-skin regime is applicable, notches are machined to be substantially long and deep. By referring to the diagram of voltage (both real and imaginary part) versus tilt angles, the depth of notches is retrieved despite the tilt effects.

## 2. Thin-Skin Regime Using Eddy-Current T-R Sensor

### 2.1. Original Method-General Formulas of Thin-Skin Regime for Self-Impedance of Tilted Coil Winding above Long Surface Crack

**Figure 1:**
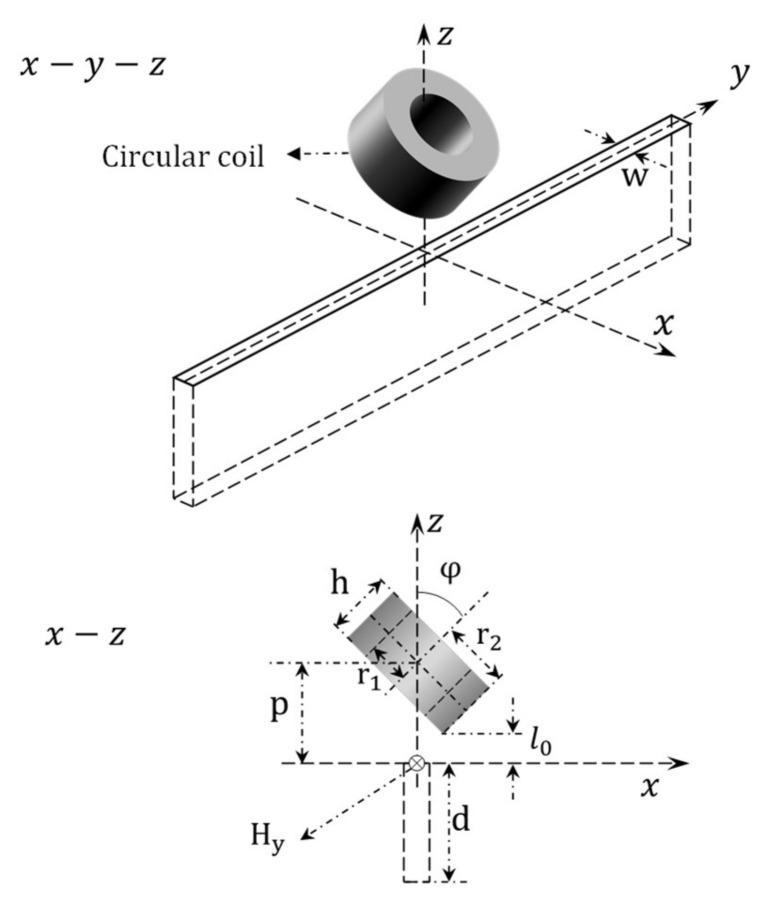
Tilted cross-sectional eddy current air-core winding above an ideal notch of conductive half-space.

The 3-D problem of a tilted cross-sectional eddy current air-core coil winding above a long surface crack slot of a conductive half-space [29] is shown in Figure 1. The sensor rotates with respect to the y axis with an angle of φ. Referring to [29], by applying Fourier transform of the magnetic field in the normal space–Cartesian coordinate system, the magnetic field intensity generated from a tilted circular coil in region $0\leq z\leq l_{0}$ above the conductive half-space without a crack is:

(1) $$H\left(x,y,z\right)=\frac{I}{2\pi }\int_{-\infty }^{\infty }\int_{-\infty }^{\infty }\frac{e^{j\left(\beta h+ux+vy\right)}\tilde{h}\left(u,v\right)}{\alpha }\left(e^{\alpha z}-{\Phi e}^{-\alpha z}\right)\left(ju\hat{x}+jv\hat{y}\right)+\left(e^{\alpha z}+{\Phi e}^{-\alpha z}\right)\hat{z}du\;dv$$

I is the excitation current flowing in the coil. j is the imaginary unit, which is expressed as the square root of −1. For the region z≤0-inside the conductive half-space without crack, the magnetic field intensity becomes:

(2) $$H\left(x,y,z\right)=\frac{I}{2\pi }\int_{-\infty }^{\infty }\int_{-\infty }^{\infty }\frac{e^{j\left(\beta h+ux+vy+\alpha_{1}z\right)}\tilde{h}\left(u,v\right)\left(1-\Phi \right)}{\alpha }\left(ju\hat{x}+jv\hat{y}+\alpha_{1}\hat{z}\right)du\;dv$$

In (1) and (2), h is the height of the coil winding. x,y, and z are axial parameters in the Cartesian coordinate system. When the test piece is non-magnetic conductive material, Β and $\alpha_{1}$ are defined as:

(3) β=usinφ+jαcosφ

(4) $$\alpha_{1}=\sqrt{\alpha^{2}+j2{\pi \sigma \mu }_{0}f}$$

σ is the electrical conductivity of test piece. $\mu_{0}$ is the vacuum magnetic permeability. f is the operation frequency of excitation currents. α is the root mean square of 2-D Fourier variables—u and v, which are related to the wavenumber of Transverse (TE) plane waves [25].

(5) $$\alpha =\sqrt{u^{2}+v^{2}}$$

In (1) and (2), $\tilde{h}\left(u,v\right)$ is the 2-D Fourier transform of the free-space magnetic field incident on z=0 at x-y plane for a unit ampere of the excitation current, which is sensor dependent. For a cross-sectional coil winding, $\tilde{h}\left(u,v\right)$ is defined as:

(6) $$\tilde{h}\left(u,v\right)=j\frac{N}{h\left(r_{2}-r_{1}\right)}\frac{M}{\beta^{3}}e^{-\alpha p}\sin\left(\frac{\beta h}{2}\right)$$

$r_{1}$ and $r_{2}$ are the inner and outer radius of coil windings, N is the turn number of coil windings. As shown in Figure 1, p and $l_{0}$ are the lift-off distance from the centre and bottom of the tilted sensor to the test piece ($p=l_{0}+r_{2}\sin\left|\varphi \right|$). M is the integral related to the first-order modified Bessel term $I_{1}$.

(7) $$M=\int_{{\beta r}_{1}}^{{\beta r}_{2}}{\tau I}_{1}\left(\tau \right)d\tau$$

To speed up the computation, the integration in (7) can be expanded as:

(8) $$M=\frac{\pi \beta }{2}\left\{r_{1}\left[I_{0}\left({\beta r}_{1}\right)L_{1}\left({\beta r}_{1}\right)-I_{1}\left({\beta r}_{1}\right)L_{0}\left({\beta r}_{1}\right)\right]-r_{2}\left[I_{0}\left({\beta r}_{2}\right)L_{1}\left({\beta r}_{2}\right)-I_{1}\left({\beta r}_{2}\right)L_{0}\left({\beta r}_{2}\right)\right]\right\}$$

$I_{n}$ and $L_{n}$ are the first-kind modified Bessel and Struve functions with order n. Empirically, according to [31], Φ is a material-dependent term and defined as:

(9) $$\Phi =\frac{\left(\alpha_{1}+\alpha \right)\left(\alpha_{1}-\alpha \right)-\left(\alpha_{1}+\alpha \right)\left(\alpha_{1}-\alpha \right)e^{2\alpha_{1}c}}{-\left(\alpha_{1}-\alpha \right)\left(\alpha_{1}-\alpha \right)+\left(\alpha_{1}+\alpha \right)\left(\alpha_{1}+\alpha \right)e^{2\alpha_{1}c}}$$

c is the thickness of test pieces. Assume the depth and gape of the long surface crack are d and w respectively. Considering the thin-skin depth regime of thick non-magnetic plates, the frequency of the current must large enough that crack depth d and length are at least three times the skin depths [30,32]. As c≫d, the sample can be treated as a non-magnetic conductive half-space. Additionally, Φ can be approximated as:

(10) $$\Phi =\frac{\alpha -\alpha_{1}}{\alpha +\alpha_{1}}$$

Referring to [29], according to the boundary condition—where the normal component of H is continuous at different material interfaces, the impedance change caused by long notches of the half-space conductor using a single tilted coil is:

(11) $$\Delta Z_{c}=\mu_{0}f\int_{-\infty }^{\infty }\frac{g\left(w\right)}{1+\frac{2v\tilde{U}\tanh\;\left(vd\right)}{\mathrm{jk}}}\frac{{\tilde{H}}_{y}\left(-v\right){\tilde{H}}_{y}\left(v\right)}{v^{2}}dv$$

In (11), $g\left(w\right)$ is a parameter dependent on the crack gape. Parameters—$g\left(w\right)$, k, $\tilde{U}$ (even functions of v) are defined in Appendix A.

${\tilde{H}}_{y}\left(v\right)$ is related to the Fourier transform of $H_{y}$ for a unit ampere of the excitation current. $H_{y}$ is the source contribution of the magnetic field intensity, which is approximated by $H\left(x,y,z\right)$ at (0,0,0) along y axis without the crack [29].

(12) $${\tilde{H}}_{y}\left(v\right)=v\int_{-\infty }^{\infty }\tilde{h}\left(u,v\right)\frac{\alpha_{1}}{a\left(\alpha +\alpha_{1}\right)}du$$

The impedance change for the non-magnetic half-space conductor without defect is:

(13) $$Z_{0}=j4{\pi \mu }_{0}\mathrm{If}\int_{-\infty }^{\infty }\int_{-\infty }^{\infty }\frac{\tilde{h}\left(-u,-v\right)\tilde{h}\left(u,v\right)\left(\alpha -\alpha_{1}\right)}{\alpha \left(\alpha +\alpha_{1}\right)}du\;dv$$

### 2.2. Method-Revised Algorithms of Mutual Impedance of Tilted T-R Sensor Scanning Cross Long Notches

**Figure 2:**
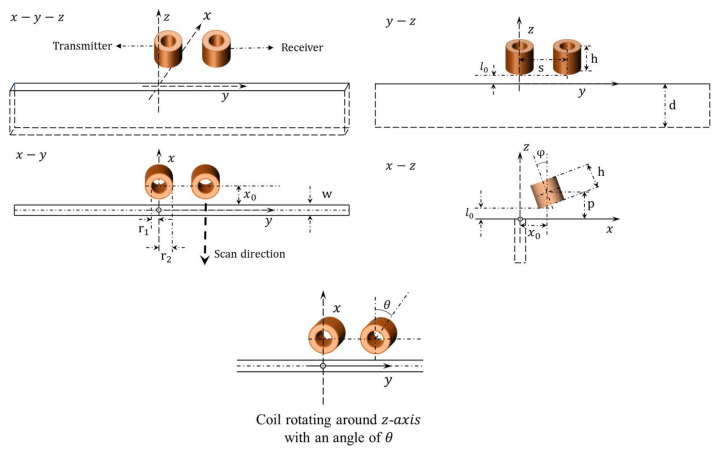
Tilted eddy current T-R sensor scanning across (x direction) and through the centre of a long surface crack slot on the conductive half-space.

In Figure 2, the T-R sensor, also termed as the driver-pickup sensor, is used for the notch depth profiling on non-magnetic metals. The transmitter and receiver are tilted-rotating with crack direction—y-axis at the same lift-off plane ($l_{0}$), with a horizontal separation distance of s. Parameters of the T-R sensor are listed in Table 1.

Assume the scan direction of tilted T-R sensor is perpendicular to the crack orientation, $x_{0}$ is the transient distance to the crack. For the transmitter, ${\tilde{H}}_{y}\left(v\right)$, H component along y axis, is related to $H_{y}$ at $\left(x_{0},0,0\right)$ without the crack. Thus, referring to (1) and (2), for the non-magnetic material, $\tilde{h}\left(u,v\right)$ becomes $\tilde{h}\left(u,v\right)e^{jux_{0}}$ when the transmitter shifted from $\left(0,0,p\right)$ to $\left(x_{0},0,p\right).$ Consequently, ${\tilde{H}}_{y}\left(v\right)$ for the transmitter becomes:

(14) $${\tilde{H}}_{yt}\left(v\right)=v\int_{-\infty }^{\infty }\frac{\tilde{h}\left(u,v\right)e^{jux_{0}}\alpha_{1}}{\alpha \left(\alpha +\alpha_{1}\right)}du$$

In addition, $\tilde{h}\left(u,v\right)$ becomes $\tilde{h}\left(u,v\right)e^{j\left(ux_{0}+vs\right)}$ when the receiver shifted from $\left(0,0,p\right)$ to $\left(x_{0},s,p\right).$ Thus, ${\tilde{H}}_{y}\left(v\right)$ for the receiver becomes:

(15) $${\tilde{H}}_{yr}\left(v\right)=v\int_{-\infty }^{\infty }\frac{\tilde{h}\left(u,v\right)e^{j\left(ux_{0}+vs\right)}\alpha_{1}}{\alpha \left(\alpha +\alpha_{1}\right)}du$$

Then, the voltage change due to notches using the tilted T-R sensor is (referring to Equation (11)):

(16) $$\Delta Z_{c}=\mu_{0}f\int_{-\infty }^{\infty }\frac{g\left(w\right)}{1+\frac{2v\tilde{U}\tanh\;\left(vd\right)}{\mathrm{jk}}}\frac{{\tilde{H}}_{yt}\left(-v\right){\tilde{H}}_{yr}\left(v\right)}{v^{2}}dv$$

Thus, the voltage change due to the notch using the T-R sensor is:

(17) $$\Delta V_{c}\left(x_{0}\right)=\mu_{0}\mathrm{If}\int_{-\infty }^{\infty }\frac{g\left(w\right)}{1+\frac{2v\tilde{U}\tanh\;\left(vd\right)}{\mathrm{jk}}}\frac{{\tilde{H}}_{yt}\left(-v\right){\tilde{H}}_{yr}\left(v\right)}{v^{2}}dv$$

In (17), $\Delta V_{c}$ is the voltage change due to the secondary magnetic field from the eddy current affected by the defect of the test piece. To mitigate the discrepancy between experimental and analytical data, the crack depth is retrieved by referring to the normalised version of (17)—$\frac{\Delta V_{c}\left(x_{0}\right)}{V_{0}}$. $V_{0}$ is the magnitude of the voltage change, which is nullified by the free space voltage to eliminate the mutual coupling effect of coils for the defect-free region of the non-magnetic plate. Referring to (13) and revised $\tilde{h}\left(u,v\right)$ for both the transmitter ($\tilde{h}\left(u,v\right)e^{jux_{0}}$) and receiver ($\tilde{h}\left(u,v\right)e^{j\left(ux_{0}+vs\right)}$), the modified $V_{0}$ for tilted T-R sensor is:

(18) $$V_{0}=\left|j4{\pi \mu }_{0}\mathrm{If}\int_{-\infty }^{\infty }\int_{-\infty }^{\infty }\frac{\tilde{h}\left(-u,-v\right)\tilde{h}\left(u,v\right)\left(\alpha -\alpha_{1}\right)e^{jvs}}{\alpha \left(\alpha +\alpha_{1}\right)}du\;dv\right|$$

By fitting the normalised analytical voltage change $\frac{\Delta V_{c}\left(x_{0}\right)}{V_{0}}$ with the measured one for different tilt angles, the depth of notches is retrieved. 

As shown in Figure 2, for the case of coil windings rotating around z-axis with an angle of θ, the free-space source term $\tilde{h}\left(u,v\right)$ is replaced with the following one.

(19) $$\tilde{h}\left(u\cos\theta +v\sin\theta ,-u\sin\theta +v\cos\theta \right)$$

## 3. Experiments

**Figure 3:**
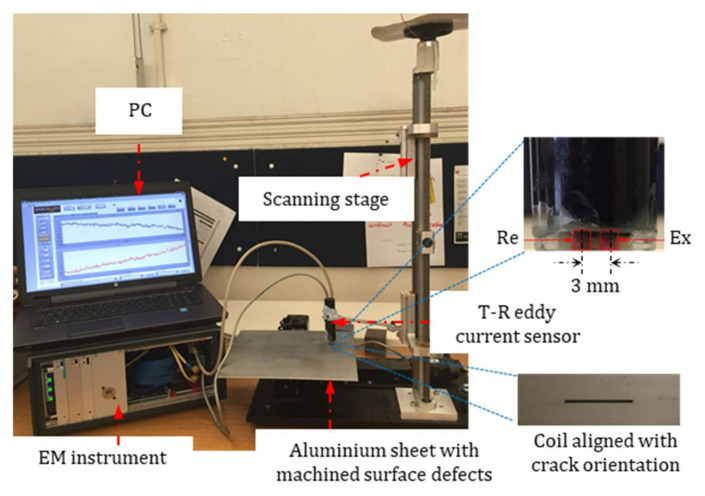
Experimental setup for electromagnetic imaging of surface cracks using T-R eddy current sensor.

As listed in Table 1, the transmitting and receiving coils are wound by enamelled copper wires. The coil windings are air-cored, with the coil wound on plastic rods by a coil winder machine. As shown in Figure 3, the T-R sensor is connected to a bespoke electromagnetic (EM) instrument to map the voltage of the T-R sensor above the specimen with machined notches. The EM instrument is fabricated by the SISP group at the School of Electrical and Electronic Engineering, University of Manchester [33,34,35]. The EM instrument is field programmable gate array (FPGA)-based, which can achieve a data frame rate of 100 k/s. The EM instrument is connected to a PC through an ethernet cable. More details of the EM instrument (including specifications, operations, features) are reported in [33]. 

**Figure 4:**
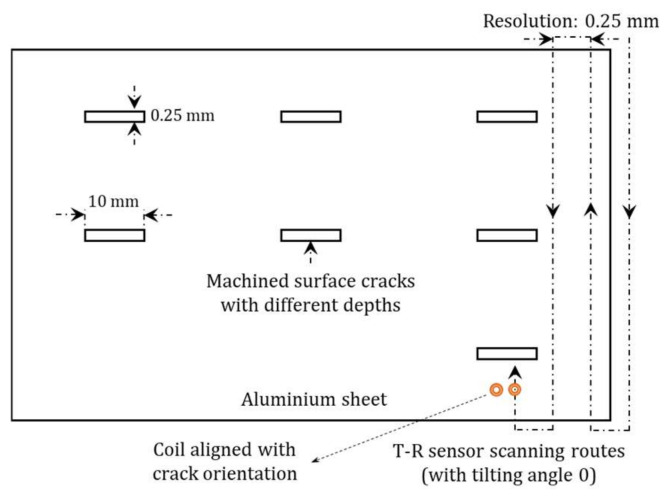
Scanning route of tilted T-R sensor for electromagnetic imaging of aluminium sheet with machined surface slots of different depths.

As illustrated in Figure 4, the sensor is controlled by a C-programmable scanning stage (Newmark Systems Inc., Chicago, IL, USA). The scanning stage is composed of two control motors, with a travel resolution of 0.2 μm per step and travel limit of 200 mm. The scanning speed of the stage is 100 mm/s. For inspection of the test piece, as listed in Table 2, the travel resolution is selected as 0.25 mm. To achieve a relatively high sensitivity of the voltage mapping, the transmitter and receiver of T-R sensor are aligned with crack orientation during the scanning. In Figure 4, a misalignment of the sensor to the crack results in a reduced sensitivity of voltage mapping. As exhibited in Table 2 and Figure 4, the aluminium sheet contains seven machined notches with identical lengths and gapes, but different depths. To ensure that the thin-skin regime valid and can be applied to the machined cracks, the (upgraded version of) EM instrument is operated under the working frequency of 300 kHz. Thus, the skin depth of the induced eddy current is 0.15 mm, which is substantially smaller than the depth of the machined slot (with a minimum depth of 0.4 mm, as shown in Table 2). 

The eddy current is designed as a driver–pickup or T-R sensor instead of a single or co-axial sensor, which is verified to have a higher spatial resolution, which doubles the resolution of single-coil sensor of the same size [36], has a wider frequency range, higher gain, and is barely affected by thermal drift [37]. Moreover, as shown in Table 1, the mean radius of the coil winding is only 1 mm, which achieves a relatively high sensitivity on the voltage mapping of notches. 

## 4. Result and Discussion

### 4.1. Scanned Voltage for T-R Sensor across Notch without Tilt Effect

**Figure 5:**
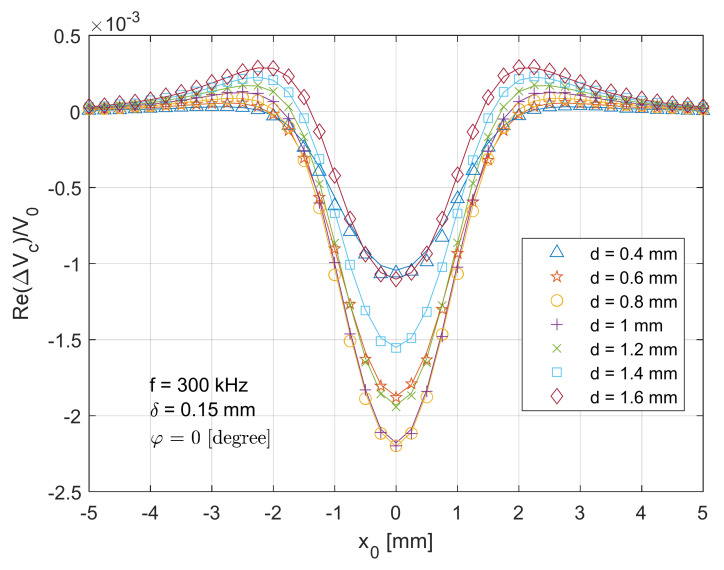
Comparison of analytical (lines) and experimental (markers) results for the real part of normalised voltage change (due to the crack) for the T-R sensor (tilt angle φ=0 degree) scanning across (x direction in Figure 2) and through the crack centre.

Figure 5 depicts a comparison between the theoretical (solid lines) and measurement (markers) data of the real part of the normalised voltage change (ReΔVcx0V0) when the T-R sensor (tilt angle φ=0 degree) scans across the centre of the crack from $x_{0}=-5$ mm (in Figure 2) to $x_{0}=5$ mm with a travelling step of 0.25 mm. It can be observed that the theoretical/analytical result fits well with the measurement, with a maximum error of around 4.4%. The sensor response (normalised voltage change) reaches its maximum magnitude when $x_{0}=0$ mm, where the T-R sensor is above the centre of the crack with a lift-off distance of 2 mm. As the T-R sensor drifts away, the T-R sensor interacts less with the test piece, which results in the attenuated (normalised) voltage change due to the crack. For $x_{0}=\pm 2$ mm, an overshoot is observed. A deeper surface slot results in a higher amplitude of the real part of voltage change. Additionally, it can be found in Figure 5 that a slightly deeper crack slot results in a higher magnitude for the real part of voltage change due to the crack. However, a further deeper crack will result in reduced signal magnitude. Intuitively, a deeper surface crack results in a higher amplitude of voltage change magnitude. However, the phase of voltage change varies with increased crack depth and different sensor positions ($x_{0}$). Thus, for some sensor positions, the real part of the voltage change does not monotonically increase with the crack depth. That is why, for the position $x_{0}=0$ mm, the results of d = 0.4 mm and d = 1.6 mm are quite similar, while for the position $x_{0}=\pm 2$ mm, the results of d = 0.4 mm and d = 1.6 mm are different.

**Figure 6:**
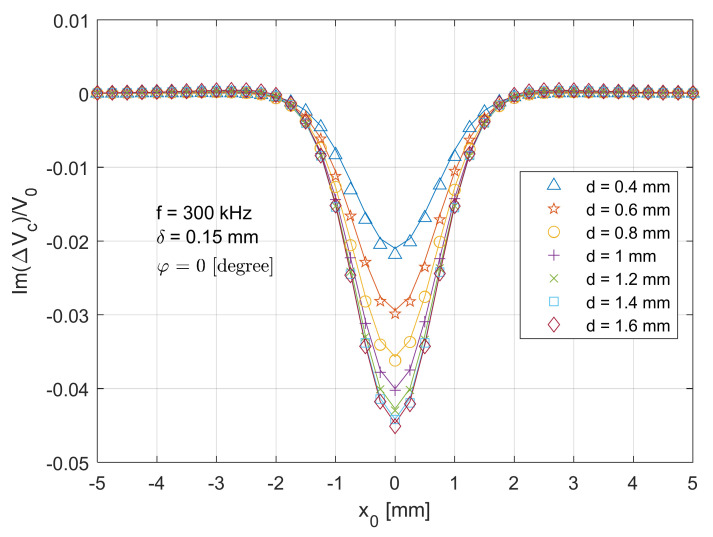
Comparison of analytical (lines) and experimental (markers) results for the imaginary part of normalised voltage change (due to the crack) for the T-R sensor (tilt angle φ=0 degree) scanning across (x direction in Figure 2) and through the crack centre.

In Figure 6, a similar symmetric trend can be found at the imaginary part of normalised voltage change (ImΔVcx0V0), when the T-R sensor scans across the centre of the crack with the same path in Figure 5. The maximum error between the analytical and experimental data is around 5.3%. Since the test piece becomes more inductive under high working frequencies (referring to [38,39,40,41,42,43,44]), the magnitude for the imaginary part of voltage changes due to the crack being much larger than (around 20 times) that of the real part. Owing to the different sensitivity, a deeper crack results in a larger magnitude of voltage change. This is because the imaginary part dominates the magnitude of the voltage change, which increase with the depth of surface crack. Compared to the real part in Figure 5, less overshoot can be observed from the imaginary part in Figure 6. More sensitivity analyses are reported in [28].

**Figure 7:**
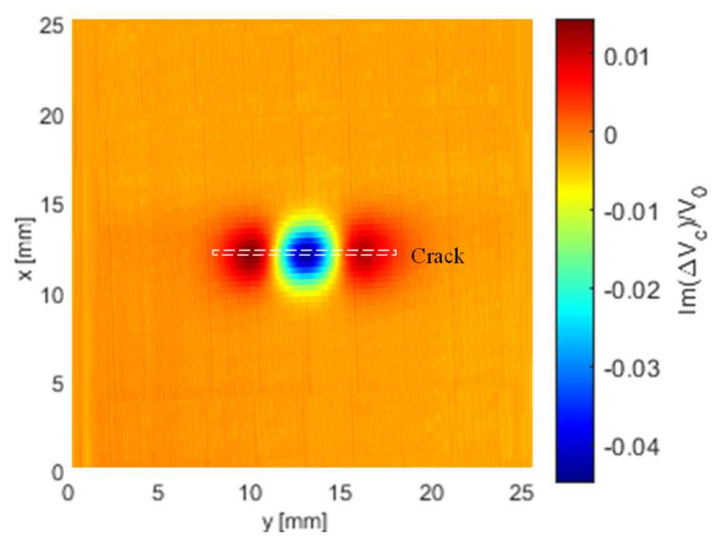
The 2-D imaging of the imaginary part of normalised voltage change (due to the crack) using the T-R sensor (tilt angle φ=0 degree) for the inspection of a surface notch with a depth of 1.6 mm.

**Figure 8:**
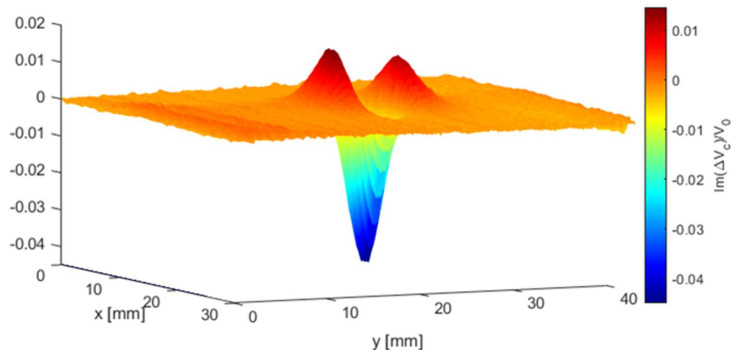
The 3-D imaging of the imaginary part of the normalised voltage change (due to the crack) using the T-R sensor (tilt angle φ=0 degree) for the inspection of a surface notch with a depth of 1.6 mm.

Figure 7 shows the 2-D voltage imaging for the imaginary part of the normalised voltage changes of the crack slot (1.6 mm deep) using the T-R sensor. The span of the plot is 30 and 40 mm (and resolution of 0.25 mm in each travel step) in the x and y directions. As can be observed from the 3-D version of (imaginary part) voltage change due to the crack in Figure 8, two bumps and one hollow exist for T-R sensor with tilt angle φ=0 degree scanning along the centre of notches in y direction. However, for the T-R sensor scanning across the crack and through its centre, only one hollow can be observed at the crack centre, which follows the same trend as in Figure 6.

### 4.2. Tilted T-R Sensor Across the Crack

**Figure 9:**
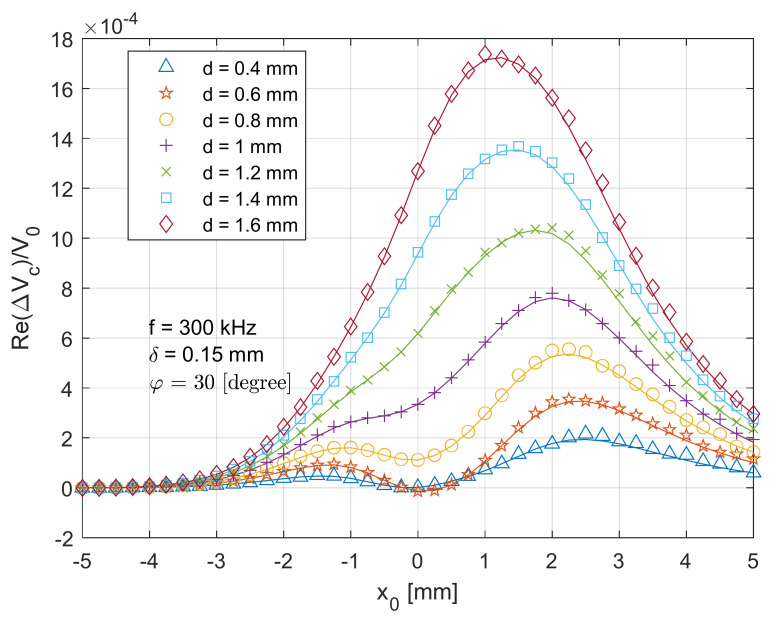
Comparison of analytical (lines) and experimental (markers) results for the real part of normalised voltage change (due to the crack) for the T-R sensor (tilt angle φ=30 degrees) scanning across (x direction in Figure 2) and through the crack centre.

**Figure 10:**
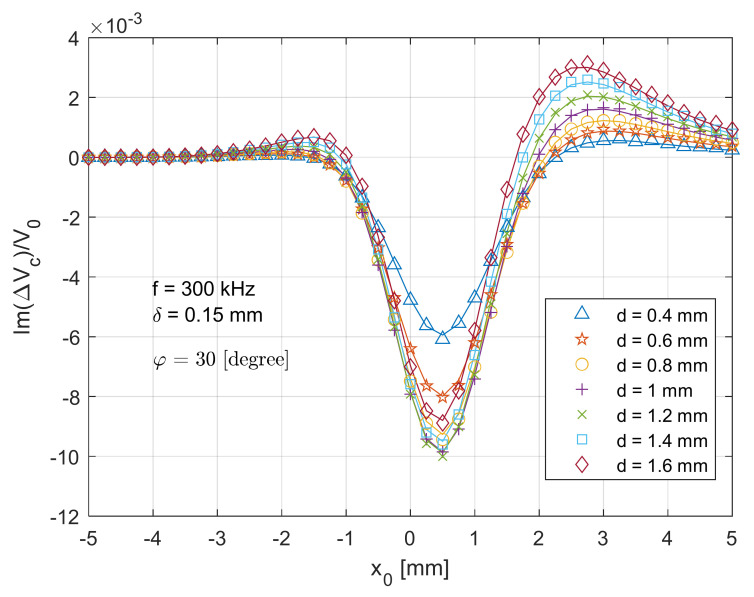
Comparison of analytical (lines) and experimental (markers) results for the imaginary part of normalised voltage change (due to the crack) for the T-R sensor (tilt angle φ=30 degrees) scanning across (x direction in Figure 2) and through the crack centre.

As shown in Figure 9 and Figure 10, when the T-R sensor rotates around the crack direction (y-axis in Figure 2) with an angle of 30 degrees, the scanned voltage for both real and imaginary part become asymmetric. The peak value point is slightly right shifted. Additionally, the real part of the scanned voltage becomes positive-dominant. Moreover, a slightly higher overshoot can be observed on the right side (around $x_{0}=2.5$ mm) of the imaginary voltage change.

**Figure 11:**
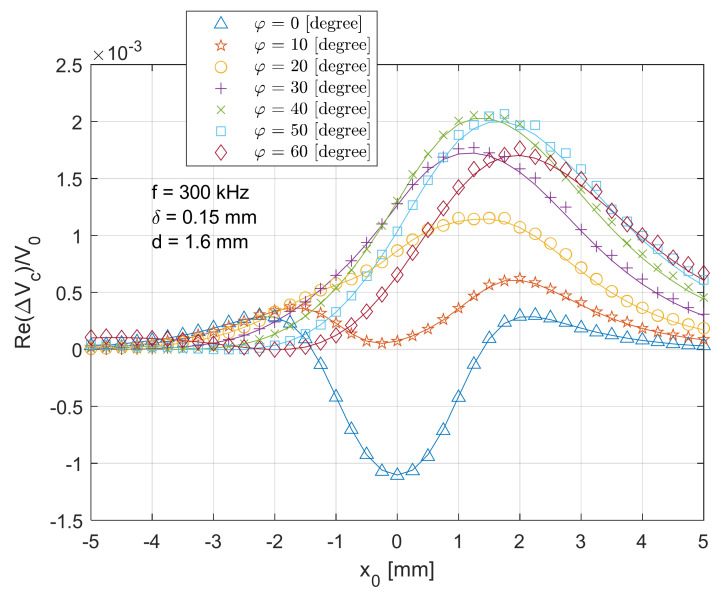
Comparison of analytical (lines) and experimental (markers) results for the real part of normalised voltage change (due to the crack) for the T-R sensor scanning across (x direction in Figure 2) and through the crack centre with the depth of 1.6 mm and different tilt angles.

**Figure 12:**
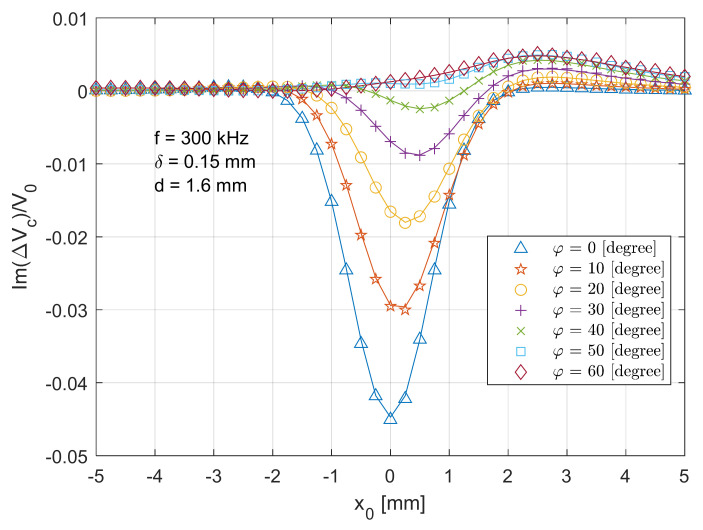
Comparison of analytical (lines) and experimental (markers) results for the imaginary part of normalised voltage change due to the crack for the T-R sensor scanning across (x direction in Figure 2) and through the crack centre with the depth of 1.6 mm and different tilt angles.

Figure 11 and Figure 12 show the real and imaginary parts of the scanned voltage for different tilt angles when the T-R sensor scans across (x direction in Figure 2) the crack centre with the depth of 1.6 mm. As the tilt angle increases, the real part of voltage changes gradually and right shifts and becomes positive-dominant. For the imaginary part of voltage change, the curve in Figure 12 also shifts right but significantly attenuates with the increased tilt angle, which is caused by the lower interactive effect or coupling effect between the T-R sensor and the defective area. As the primary magnetic field is lack of symmetry with respect to z-axis when the coils are tilted, the induced eddy current also becomes asymmetric [29]. As a result, the secondary magnetic field from the induced eddy current and the overall received signal from the probe become less symmetric with respect to the crack centre. Thus, the voltage curves in Figure 11 and Figure 12 become asymmetric with respect to the position $x_{0}=0$ mm. As explained regarding Figure 5, the phase of voltage change varies with crack depth. Since the imaginary part dominates in the magnitude of voltage change, in Figure 12, a deeper crack results in a higher overall amplitude of voltage change.

### 4.3. Retrieval of Surface Crack Depth

**Figure 13:**
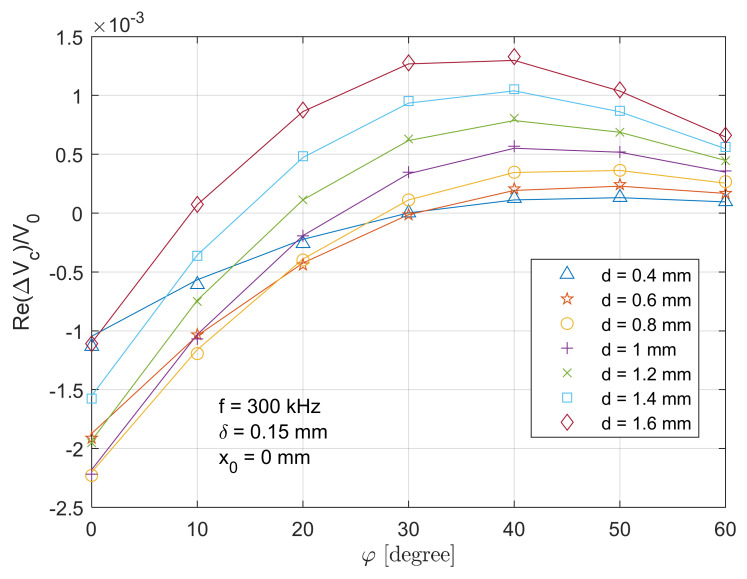
Comparison of analytical (lines) and experimental (markers) results for the real part of normalised voltage change (due to the crack) versus tilt angles when the T-R sensor is above the crack centre ($x_{0}=0$ mm).

**Figure 14:**
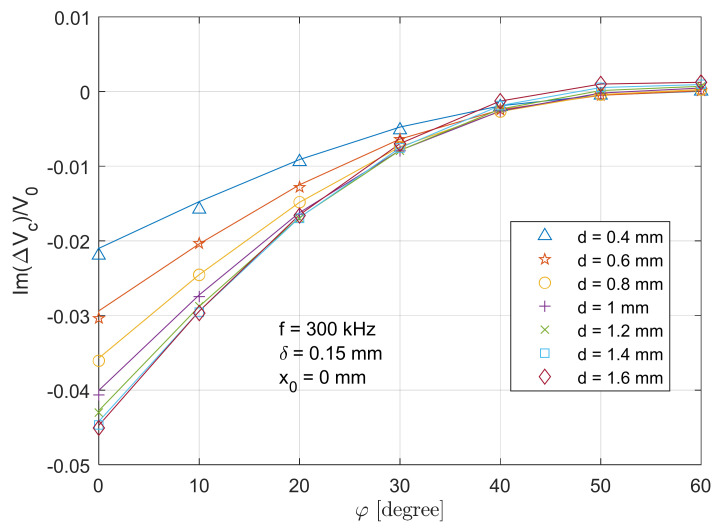
Comparison of analytical (lines) and experimental (markers) results for the imaginary part of normalised voltage change (due to the crack) versus tilt angles when the T-R sensor is above the crack centre ($x_{0}=0$ mm).

**Figure 15:**
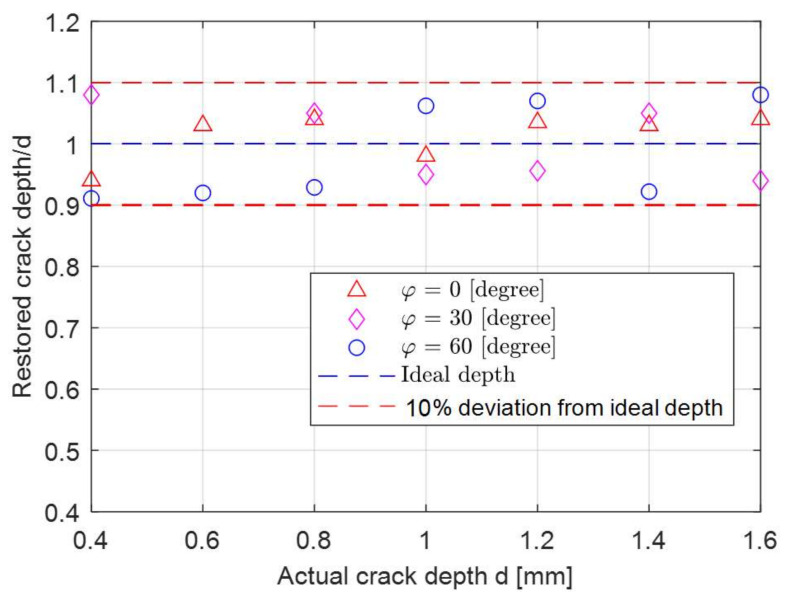
Retrieval of the surface crack slot versus actual depths for the tilt angles of 0, 30, and 60 degrees.

The voltage change for T-R sensor above the crack centre has a decent amplitude and sensitivity (particularly for a small tilt angles), which is used to retrieve the notch depth considering different tilt angles of the T-R sensor. Figure 13 and Figure 14 depict the real part and imaginary parts of the voltage change versus tilt angle of sensors at different crack depths. The analytical result exhibits a decent agreement with the measured ones, with a maximum error of 4.1% and 5.3% for the depth of 0.4 mm case. Figure 15 illustrates the retrieval of crack depth versus its actual value under different tilt angles. In Figure 15, the blue dash line with a unit constant shows the ideal depth by means of dimensional measurement. Different markers depict the retrieved thickness from the proposed method at different tilt angles. Retrieval and measurement are conducted at crack depths from 0.4 mm to 1.6 mm with a uniform increment of 0.2 mm. The uncertainty of retrieved depth and depth by means of dimensional measurement is 0.02 mm and 0.01 mm. By altering parameter d when fitting the analytical value (both the real and imaginary part) of $\frac{\Delta V_{c}\left(x_{0}\right)}{V_{0}}$ (Equations (17) and (18)) with the measured one, the deviation of notch depth profiling is controlled within 10% for all the crack slots and tilt angles. The maximum deviation (9%) of the retrieved depth occurs at a crack depth of 0.4 mm with a tilt angle of 60 degrees, where the crack depth is 2.67 times that of the skin depth (which is slightly below the criteria of a thin-skin regime). Overall, it can be observed that a larger tilt angle results a large deviation of depth retrieval.

## 5. Conclusions

In this paper, based on the thin-skin regime, an impedance or voltage algorithm for tilted T-R sensor scanning across notches has been proposed for the first time. By referring to both real and imaginary parts of the diagram of voltage versus tilt angles, the depth of notches is retrieved despite of tilt effects. Experimental voltage imaging has been conducted for the eddy current T-R sensor scanning over notches on an aluminium sheet with different tilt angles-circumferential tilt regarding to crack orientation. From the result, the error of depth profiling for notches has been controlled within 10%. However, the proposed method merely analysed the circumferential tilt effect of the T-R sensor regarding to crack orientation (θ=0 in Equation (19)). The longitudinal tilt effect of the T-R sensor on the notch depth profiling will be investigated in the future. In addition, the proposed algorithms are based on the non-ferrous metals. Further investigations of notches on ferrous steel will be carried out in the future.

## Figures and Tables

**Table 1:** Parameters of the T-R eddy current sensor and measurement setup.

Parameter	Transmitter Coil or Receiver Coil
Inner radius $r_{1}$ (mm)	0.75
Outer radius $r_{2}$ (mm)	1.25
Turns N	300
Spacing s (mm)	3.0
Coil wire diameter (mm)	0.071
Coil height h (mm)	3.0
Lift-off $l_{0}$ (mm)	2.0
Tilt angle φ (degree)	0:10:60
Working frequency f (kHz)	300
Magnitude of free-space voltage (152 kHz) (V)	1.12
Driven current (mA rms)	48
Free-space coil inductance (H)	$2.45\times 10^{-5}$
Free-space coil DC resistance (Ω)	$3.32\times 10^{-8}$

**Table 2:** Parameters of the conductive samples with long surface notch.

	Parameter		Value	
Aluminium sheet	Electrical conductivity σ (MS/m)		36.9	
	Relative permeability		1	
	Thickness (mm)		2.0	
	Magnitude of voltage change $V_{0}$ (mV) without surface notches under 300 kHz		225.7	
	Skin depth (mm) under 300 kHz		0.15	
Machined surface crack slot	Width/gape w (mm)		0.25	
	Length (mm)		10.0	
	Depth d (mm)		0.4:0.2:1.6	

## Data Availability

Not applicable.

## Appendix A

Followings show the definition of parameters $g\left(w\right)$, k, and $\tilde{U}$ in (11),

(A1) $$g\left(w\right)={\mathrm{jg}}_{f}w+\left(1+j\right)\delta \left(g_{f}-\frac{g_{s}w}{2}\right)+\frac{g_{k}\delta^{2}}{2}$$

δ is the skin depth function of the non-magnetic materials.

(A2) $$\delta =\sqrt{\frac{1}{{\pi \sigma \mu }_{0}f}}$$

In (A1),

(A3) $$g_{f}=v\tanh\;\left(vd\right)$$

(A4) $$g_{s}=\left[1+\frac{2v}{\mathrm{jk}}\left(\tilde{U}+\tilde{V}\right)\tanh\left(vd\right)-\mathrm{sech}\left(vd\right)\right]v^{2}$$

(A5) $$g_{k}=\left\{\mathrm{sech}\left(vd\right)-\frac{8}{\pi }\left[1+\frac{v}{\mathrm{jk}}\left(\tilde{U}+\tilde{V}\right)\tanh\left(vd\right)-\mathrm{sech}\left(vd\right)\right]\right\}v^{2}$$

In (A5), $\tilde{U}$ and $\tilde{V}$ are even functions. For the non-magnetic materials, $\tilde{U}$ and $\tilde{V}$ are defined as,

(A6) $$\tilde{U}=\frac{1}{2\pi }\left[\frac{1}{\varepsilon }\ln\left(\frac{1+\varepsilon }{1-\varepsilon }\right)-\frac{1}{\sqrt{1+\varepsilon^{2}}}\ln\left(\frac{1+\sqrt{1+\varepsilon^{2}}}{1-\sqrt{1+\varepsilon^{2}}}\right)\right]$$

(A7) $$\tilde{V}=-\frac{1}{\pi }\ln\left(\frac{\varepsilon }{\sqrt{\varepsilon^{2}-1}}\right)$$

In (A5), k is defined as,

(A8) $$k=\frac{-1+j}{\delta }$$

In (A6) and (A7),

(A9) $$\varepsilon =\frac{v}{k}$$
